# Can Checklists Solve Our Ward Round Woes? A Systematic Review

**DOI:** 10.1007/s00268-022-06635-5

**Published:** 2022-07-03

**Authors:** Ellie C. Treloar, Ying Yang Ting, Joshua G. Kovoor, Jesse D. Ey, Jessica L. Reid, Guy J. Maddern

**Affiliations:** 1 College of Medicine and Public Health, Flinders University, Adelaide, Australia; 2 Discipline of Surgery, The University of Adelaide, The Queen Elizabeth Hospital, 28 Woodville Road, Woodville, SA 5011 Australia

## Abstract

**Background:**

Accurate and thorough surgical ward round documentation is crucial for maintaining quality clinical care. Accordingly, checklists have been proposed to improve ward round documentation. This systematic review aimed to evaluate the literature investigating the use of checklists to improve surgical ward round documentation.

**Methods:**

MEDLINE, EMBASE, and PsycINFO were searched on August 16, 2021. Study selection, data extraction, and risk of bias assessment were performed in duplicate. We included English studies that investigated the use of checklists during ward rounds in various surgical subspecialties compared to routine care, where the rates of documentation were reported as outcomes. We excluded studies that used checklists in outpatient, non-surgical, or pediatric settings. Due to heterogeneity of outcome measures, meta-analysis was precluded. This study was registered with PROSPERO (ID: CRD42021273735) and followed the Preferred Reporting Items for Systematic Reviews and Meta-Analyses 2020 (PRISMA 2020) reporting guidelines.

**Results:**

A total of 206 studies were identified, only 9 were suitable for inclusion. All included studies were single-center observational studies, spanning across seven surgical specialties. Rates of documentation on 4–23 parameters were reported. Documentation for all measured outcomes improved in 8/9 studies; however, statistical analyses were not included. There was a high risk of bias due to the nature of observational studies.

**Conclusion:**

Ward round checklists can serve as a useful tool to improve inpatient care and safety. Currently, there is no high-level evidence showing the effectiveness of checklists on ward round documentation. The synthesis of results indicates that further high-quality research is imperative.

**Supplementary Information:**

The online version contains supplementary material available at 10.1007/s00268-022-06635-5.

## Introduction

Ward rounds are fundamental components of in-hospital surgical care. Usually, at least once daily, the treating team will visit each patient under their care. Surgical consultants, fellows, registrars, and students will be joined by nurses, allied health professionals, and other staff involved in patient care. Typically led by a senior doctor, there will be a review of patient progress, refinement of diagnosis, initiation of treatment, and discharge planning [1], while junior doctors document the discussion in paper case notes or electronic medical records [2–4].

Ward round quality and documentation can influence patient outcomes [3, 5], and the quality of postoperative care can have more of an effect on surgical outcomes than the operation itself [6]. Ward round documentation serves as a communication tool that facilitates the continuity of care and is often the only correspondence between treating teams [7]. Poor communication between clinical teams can lead to patient complications [8–11], thus it is imperative that ward round documentation is thorough to ensure the highest quality of patient care [12].

Surgical teams are routinely under time pressure [13], and as a result ward rounds can become unstructured, highly variable, and rushed, leading to poor documentation and the potential to overlook important aspects of patient care [5, 14, 15]. To ensure a consistent standard of care, the implementation of standardized checklists can be effective [16, 17]. Ward round checklists are defined as a pre-determined list of items and agendas to be carried out and checked daily. They outline the tasks that are required and the order they are to be performed [18, 19]. Checklists may be useful for rotational members that may not be familiar with the unit protocols, during handover periods, and periods of decreased staffing, such as holidays and weekends. However, the effectiveness of ward round checklists for improving surgical ward round documentation is unclear. This systematic review aims to investigate the impact of surgical ward round checklists on the rates of documentation.

## Methods

This systematic review was registered with PROSPERO (ID: CRD42021273735) and followed the Preferred Reporting Items for Systematic Reviews and Meta-Analyses (PRISMA) reporting guidelines [20]. Research questions were framed using the PICO guidelines [21]. The population was doctors caring for surgical inpatients. The intervention was any checklist used in an inpatient multidisciplinary ward round setting. When reported, the comparator comprised ward rounds performed without a checklist. Outcomes of interest were rates of documentation as reported by the authors such as: history, examination, venous thromboembolism (VTE) assessment, antibiotic stewardship, dietary plan, estimated discharge, and subjective measures. Exclusion criteria included checklists used for non-surgical ward rounds, checklists used in the outpatient setting, and studies that reported results for less than 20 patients. Abstracts and poster presentations were excluded, as well as non-English studies.

### Search strategy

A systematic literature search was performed on August 16, 2021, using MEDLINE, EMBASE, and PsycINFO databases. The search was restricted to peer-reviewed studies published after January 1, 1980. Preliminary searches did not identify any suitable studies prior to 1993. However, the authors agreed upon the date restriction of 1980 to ensure the search remained comprehensive. Searches were designed with input from a biomedical research librarian. Key search terms were: [(ward round* or clinical round* or teaching round* or patient round*).mp. OR Teaching Rounds/AND Documentation/OR (document* or template* or checklist* or proforma* or check-list* or ticklist* or tick-list*).mp. AND exp Specialties, Surgical/ OR (surgery or post-operative or postoperative).mp. AND limit 10 to yr = “1980 –Current”]. The full search strategy can be viewed in supplementary material (Supplementary Fig. 1).

### Study selection

Two authors (ET and YT) independently screened titles and abstracts generated from the systematic search. This was facilitated using standardized pre-piloted forms via web application (Covidence; Veritas Health Innovation, Melbourne, VIC, Australia). Full-text articles deemed to be relevant on title and abstract screening were reviewed by both authors independently. Any disagreements were resolved by a third reviewer (JE). Reference lists of included studies were screened for suitable studies.

### Data extraction

A pre-filled form was used to extract data from included studies. Two authors (ET and YT) extracted the data to populate the form. Findings were compared and disagreements were resolved by discussion. Fields on the form included first author, year of publication, journal, country, study period, type of study, aim, number of patients, cohort, specialty, interventions, style of checklist/proforma, safety parameters measured, outcomes, strengths/weaknesses, control, and other.

### Data analysis

Extracted data were synthesized in narrative and tabular formats. Study outcomes were analyzed in qualitative and quantitative fashion. Meta-analysis was precluded due to significant heterogeneity in included study endpoints. The Newcastle–Ottawa scale was used to assess methodology quality of the included non-randomized observational studies [22]. The authors independently (ET, YT) assessed the data and then compared their results, any disagreements were resolved via discussion. Attempts were made to obtain missing data by contacting the corresponding author.

## Results

### Search results

**Fig. 1:**
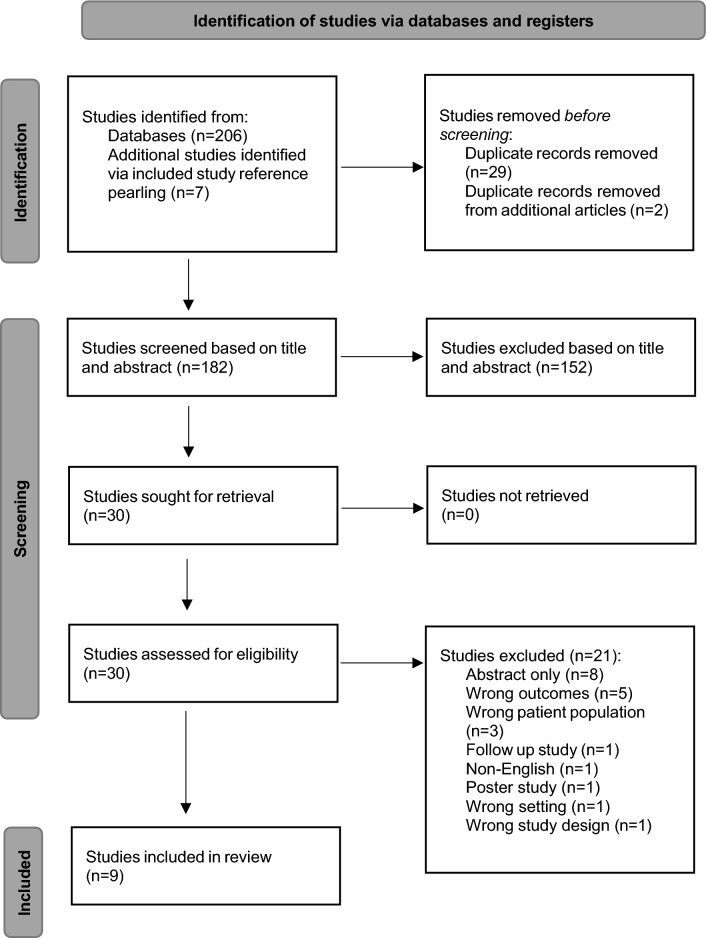
PRISMA flow diagram

A flow diagram outlining the results of study selection is shown in Fig. 1. The systematic search found a total of 206 records (158 on EMBASE and 48 on MEDLINE), with 7 additional studies found through reference pearling. After duplications were removed, a total of 182 articles were determined suitable. Of these, 152 articles were excluded, leaving 30 articles for full-text review. Studies that were included for full-text review but were excluded due to not meeting inclusion criteria can be found in supplementary material (Supplementary Table 1). After selection criteria were applied, 9 articles were deemed suitable for inclusion and analysis.

### Study findings

Nine studies were included in this systematic review. The oldest study was published in 2011, the most recent in 2019. These studies were conducted in the United Kingdom (*n* = 5), Australia (*n* = 2), Ireland (*n* = 1), and New Zealand (*n* = 1). The specialties included in the studies were general surgery (*n* = 4), vascular surgery (*n* = 2), plastic surgery (*n* = 1), acute surgery (*n* = 2), urology (*n* = 2), neurosurgery (*n* = 1), and orthopedic surgery (*n* = 1). All included studies were single-center studies: eight were pre- and post-intervention observational studies and one described both pre- and post-intervention and cross-sectional results. Study periods varied from 8 days to 3 years. A summary of the characteristics of the included studies can be viewed in Table 1, and the full table can be viewed in supplementary material (Supplementary Table 2).

**Table 1:** Summary of included studies characteristics

Author, Year	Study Period	Participants	Blinding	Intervention	Comparator
Al-Mahrouqi H et al., 2013 [23]	6 months	5–6 Consultant surgeons, Registrars, and House Officers in each arm	Blinded	Sticker-based proforma with free-text space and checklist selection to be attached to ward round notes	Routine-care
Banfield D et al., 2017 [27]	3 years	Not stated	Unblinded	Laminated checklist kept in handover office	Routine-care
Dhillon P et al., 2011 [24]	8 days	5 consultant-led teams	Unblinded	Sticker-based proforma with free-text space and circle selection to be attached to ward round notes	Routine-care
Dolan R and Broadbent P, 2016 [7]	1 week	Not stated	Unblinded	Printed ward round proforma placed in patient notes with free-text space and circle selection	Routine-care
Gilliland N et al., 2018 [26]	3 months	Not stated	Unblinded	Printed ward round proforma placed in patient notes with free-text space and circle selection	Routine-care
Krishnamohan N et al., 2019 [3]	8 months	Not stated	Blinded	Sticker-based checklist with tick boxes and free-text space to be attached to ward round notes	Routine-care
Ng J et al., 2018 [25]	6 months	Not stated	Unblinded	Cycle 1: Sticker-based proforma with free-text space and circle selection to be attached to ward round notes Cycle 2: Sticker-based checklist with tick boxes and free-text space to be attached to ward round notes	Routine-care
Pitcher M et al., 2015 [14]	4 weeks	Not stated	Blinded	Printed ward round proforma placed in patient notes with free-text space and tick boxes	Routine-care
Talia A et al., 2017 [11]	4 weeks	Four junior medical staff, consistent in both arms	Blinded	Printed ward round proforma placed in patient notes with free-text space and tick boxes	Routine-care

Checklist format varied across the studies. In four studies, proforma stickers were inserted directly into case notes by the investigators [3, 23–25]. These were small, checklist style stickers providing guidance about what to include in a ward round (such as estimated discharge date). Four studies used loose paper that clinicians directly wrote on, and these became part of the case note and patient record [7, 8, 14, 26]. The final study used a laminated poster that was displayed in the office but not on the ward [27]. Three of the paper-based checklist studies involved ‘Plan, Do, Study, Act’ (PDSA) cycles, where the checklist was amended after each round [25–27]. All included studies used paper-based case notes.

A range of 4–23 outcomes were reported in the included studies, with the most common being: ‘observations/vitals’ (*n* = *6/9*), ‘management plan’ (*n* = 5/9) ‘dietary plan’ (*n* = 5/9), ‘signature’ (*n* = 4/9), ‘examination’ (*n* = 4/9), ‘fluid balance/ prescription’ (*n* = 4/9), ‘VTE assessment’ (*n* = 4/9), and ‘impression’ (*n* = 4/9). Overall, there were 40 distinct outcomes measured across the nine studies. Of these, 25 outcomes were reported in more than one study (summarized in Table 2). The full table for all outcomes measured can be viewed in supplementary material (Supplementary Table 3). Overall, observations/vitals, review/request of blood test results, fluid balance (including prescription of intravenous fluids), and VTE assessment, all reported the biggest increases in documentation compliance. Only two studies reported overall documentation compliance, which increased in all outcomes (18–53%) [3, 23]. In one study, ‘Signature’ [25] was the only outcome that decreased in documentation compliance, but this was not statistically significant. Improvement in documentation for all but one outcome [25] was found in three studies [8, 23, 25]. Despite this, slightly less than half (47%) of the outcomes measured were statistically significant. Five other studies also demonstrated an improvement in documentation compliance in all outcomes; however, statistical significance was not reported [3, 7, 13, 26, 28].

**Table 2:** Outcomes showing changes in rates of documentation after checklist implementation

	Outcomes	Al-Mahrouqi 2013 [23]	Banfield 2017 [28]	Dhillon 2011 [24]	Dolan 2016 [7]	Gilliland 2018 [26]	Krishnamohan 2019 [3]	Ng 2018 [25]	Pitcher 2015 [27]	Talia 2017 [11]
Details	Date & Time	35%						2%		
	Team name			57%				5%		
	Signature	2%			4%			−5%		
	Grade				38%			57%		
	Presence of nurse				38%			65%	78%	
Subjective	History				67%					34%
	Subjective				16%					34%
Objective	Examination				52%			39%		18%
	NEWS					93%		42%		
	Observations/vitals			46%		36%	53%	36%	53%	6%
	Cannulas							88%	74%	
	Wounds/drains								44%	32%
	Antibiotics					100%	39%	77%	39%	
	Bloods Test Review/request			70%			57%	51%	57%	
	Drug chart review						60%	74%	60%	
	Fluid balance/prescription					79%	68%	85%	68%	
	Bowel chart								24%	50%
	VTE assessment					63%	46%		46%	85%
Assessment	Impression	21%	27%		52%			48%		
Plan	Management plan			54%	2%			2%		5%
	Dietary plan	10%			67%			50%		61%
	EDD				20%				48%	
Overall		18%					53%		53%	

### Risk of bias

A summary of the risk of bias of the studies is presented in Table 3, and the full risk of bias assessment can be found in supplementary material (Supplementary Table 4). Included studies scored between three and six out of a possible nine stars. Three out of the nine studies were blinded.

**Table 3:** Risk of bias assessment using the Newcastle–Ottawa scale

Study	Selection	Comparability	Outcome	Total
Al-Mahrouqi H et al., 2013 [23]	2	1	3	6
Banfield D et al., 2017 [27]	0	0	3	3
Dhillon P et al., 2011 [24]	3	0	3	6
Dolan R and Broadbent P, 2016 [7]	1	0	3	4
Gilliland N et al., 2018 [26]	1	0	3	4
Krishnamohan N et al., 2019 [3]	1	0	3	4
Ng J et al., 2018 [25]	1	0	3	4
Pitcher M et al., 2015 [14]	1	1	3	5
Talia A et al., 2017 [11]	2	1	3	6

## Discussion

The nine included studies were heterogenous in study design, interventions used, and outcomes measured. All studies used paper-based notes/checklists. There was a high risk of bias in the included studies. While each study used a unique checklist intervention, eight common outcomes were measured in four or more studies. Despite the positive trend of improved documentation, only one study described the impact on patient outcomes, where errors in prescription, antibiotic use, fluid balance, patient observations, and the number of diagnosed VTE cases were reduced with the use of a checklist [3]. Few studies assessed the effect of ward round checklists on patient outcomes, these include improved patient-perceived quality of care as well as reduced errors in prescription [29, 30]. From this review, the authors were unable to determine which individual items on the checklist had an impact on patient outcomes. As the primary function of the ward round is to create and set in motion plans for patients, it would be of importance that details regarding management plans are accurately documented. Future studies could be designed to investigate if checklists have an impact on the documentation of management plans and whether this documentation affects patient outcomes. Although the study population targeted doctors conducting ward rounds for surgical inpatients, the consistency of teams or individual doctors was described in only 1/9 studies [7], which reduces the strength of the results.

The introduction of the World Health Organization’s (WHO) Surgical Safety checklist is undoubtedly one of the most prominent successes of implementation of a checklist in medicine [31]. This preoperative checklist was introduced in 2008 and has been successful in reducing surgical complications and mortality [17, 31]. Checklists help focus cognitive efforts on complex problems rather than wasting it on routine tasks and they ensure critical tasks are not skipped, whether due to memory, or a false sense of security [32]. Previous systematic reviews looking at checklist implementation show benefits to patient safety without impacting quality of care [33, 34]. Checklists improve documentation compliance, understanding of daily goals [35, 36], perception of teamwork [37], and patient outcomes across various clinical settings [33, 34]. Additionally, checklist use has demonstrated to decrease length of stay [36, 38], reduce critical information loss [39], technical errors [40], and overall adverse outcomes [41].

Many surgical errors occur outside the operating room (53–70%) [42–44]. This highlights a gap in the areas of health care such as the hospital ward round. The UK General Medical council proposed recommendations and guidelines for good documentation practice which include documenting clinical findings, decisions made, information given, any drugs provided to the patient, and who wrote the record [25, 45]. A popular structure of the ward round that adheres to these guidelines is the use of headings ‘Subjective, Objective, Assessment, and Plan,’ or simply known as SOAP [7]. Limitations to using SOAP include brevity, order of the acronym, and inability to integrate time into its framework [46]. Despite the limitations, the acronym assists good diagnostic reasoning and provides an easy-to-follow guide to documentation [46]. Recommendations from The Royal College of Physicians and The Royal College of Nursing highlight the importance of checklists for minimizing medical errors, ensuring thorough documentation, and the promotion of cost-effective strategies to timely discharge [47]. Current literature suggests that checklists can improve patient documentation and effective communication in hospitals [3, 48].

### Perception

Checklists were found to be useful for learning, as a guide to documentation, and contributed positively to patient safety outcomes [3, 27]. Checklists serve as a platform to empower junior doctors to ask important questions regarding patient care in an otherwise intimidating environment [27]. The overall perception of ward round checklists (from this systematic review and previous studies) is positive [30, 49–53]. However, some clinical staff are hesitant incorporate checklists into the ward round as they feel it will increase time spent on the ward round [54]. Hence, a checklist that would encourage long-term use would need to be easy to implement and have robust evidence regarding improvement in patient safety [17].

### Quality of documentation

The primary goal of a ward round checklist is to ensure observations and discussions are recorded accurately and efficiently, yet the quality and accuracy of the documentation were not assessed in any of the included studies. There is limited literature regarding how accurately ward round documentation reflects the events/discussion that occurred during the ward round. A concern regarding the use of checklists is that it may propagate copy and pasting of patients’ medical records, especially when records are kept electronically [55]. Documentation of a plan does not reflect that the plan has been carried out. Such can occur in the prescription of medications and intravenous fluids, where administration nor appropriateness are documented. This may be reflective of one study [3], where despite the increase in the rates of documentation of VTE prophylaxis prescription, the number of diagnoses of VTE cases did not decrease dramatically (11 vs. 10). This may have occurred due to errors in prescription or in delivery. There may be other confounding factors, but this highlights that a checklist can act as a useful tool to prompt consideration and improve documentation. The included studies did not measure the quality of documentation, discussions in the ward round, nor the implementation of plans put in place. Further studies should be conducted to investigate how well the events of the ward rounds are being documented, and further, if a checklist improves the accuracy of correct documentation.

### Training/education

Education and training on how to use the checklist varied in each study. Two studies provided training to doctors on how to use the checklist during the ward rounds [3, 28]. Three studies provided oral presentations to staff, emphasizing the importance of ward round documentation [8, 27, 28]. Two studies allocated 1 week to 6 months for doctors to practice using the checklist before documentation was audited [7, 23].

### Cost

The cost of checklist implementation may be associated with printing and stationery, which could cause adoption hesitancy. Cost of stickers is approximately £0.10/patient/day [25], which is a relatively small proportion of the total cost of patient in-hospital stay. The National Institute for Health and Care Excellence (NICE) guidelines have indicated that a checklist is the most cost-effective strategy to promote timely discharge, and it is likely that the benefits would outweigh the costs [56]. There are no studies that show checklists in ward rounds reduce healthcare costs; however, it could be inferred that improvements in documentation could reduce the costs associated with medication errors and medicolegal issues. Additionally, with increasing uptake of electronic medical records in hospitals, costs may be of less relevance when checklists are provided electronically. Electronic delivery of checklist may also help uptake, especially if integrated directly into electronic health records.

### Time limitations

The use of a checklist during ward rounds may inadvertently lead to longer ward rounds due to the meticulous process. However, two out of three studies reporting on time taken during ward rounds demonstrated a reduction in time taken when checklist was used [7, 28]. Other studies that have implemented checklists during ward rounds have shown that there was either no difference or less time spent on ward rounds [5, 53, 57, 58]. Time limitations should not be an issue when checklists are designed well. Checklists bring order to an otherwise unstructured process and with practice and their use should improve efficiency while ensuring patient safety and outcomes.

### Long-term usage

Five studies reported the rates of checklist uptake (between 53 and 79%) [3, 23, 25–27]. These post-intervention measurements of compliance occurred between 1 and 6 months after baseline [23, 26]. Two studies reported on the uptake of ward round checklists after the initial periods of study, with follow-ups ranging between 3 months and 2 years, where compliance was maintained between 72 and 75% [3, 27]. In one of these studies, follow-up compliance only dropped 3% in 3 months [3], and the other indicated that at 22 months documentation compliance was higher than the initial study [27]. Despite the drop in one study, overall rates of documentation were still observed to be higher than prior to introduction of checklists. The reason for decrease in usage of checklists was not ascertained from the included studies, but this is likely due to the end of the initial phase of study where the usage of checklist was not emphasized. It is encouraging that there were adopters of checklists, suggesting implementation is feasible and valuable to ward rounds. Alamri et al. investigated documentation rates 2 years after introduction of the checklist and found both improvement and regression of documentation rates of various outcomes [12]. As evidence around this area grows, the role and the use of checklists in ward rounds will be better defined.

## Strengths and limitations

The strengths of this review are the wide scope of different specialties, methods, and outcomes that are prevalent in the nine studies. Seven different surgical specialties, three types of intervention (proforma/template/sticker), varying levels of checklist education, follow-up, and blinding, mean that we could evaluate the effect checklists have on ward round documentation in many different environments. The strengths of the study were limited mainly by the study design of available literature. For example, all included studies were paper based. Given the rapid uptake of electronic records, it is vital that there is more research on optimizing e-documentation, particularly in the ward round setting. As this was a review of observational studies, there was an innate risk of bias in all studies. Due to the scarcity of literature regarding the use of checklist in ward rounds and patient outcomes during initial search, a meta-analysis would not be useful. Future research should be conducted in settings utilizing electronic checklists, whether incorporated into health records or not, and should investigate the rates of documentation against patient outcomes such as mortality and morbidity. At the same time, critical data in ward notes that would affect patient outcomes could also be investigated. These will result in higher-level evidence on the use of checklists in ward rounds.

## Conclusion

The current literature suggests that implementation of a checklist in the surgical ward round benefits and increases documentation rates. Checklists were also found to be perceived positively by clinicians and they do not make ward rounds longer. However, there is no high-level evidence showing the effectiveness of checklists on ward round documentation. Ward round checklists have potential to improve inpatient care, thus future research should comprise studies of more robust design evaluating the effectiveness of checklist implementation and patient outcomes. Additionally, the accuracy of surgical ward round documentation should be investigated in similar fashion.

## Supplementary Information

Below is the link to the electronic supplementary material.

Supplementary file1 (DOCX 14 kb)

Supplementary file2 (DOCX 17 kb)

Supplementary file3 (DOCX 34 kb)

Supplementary file4 (DOCX 23 kb)

Supplementary file5 (DOCX 17 kb)

## Abbreviations

VTE: Venous thromboembolism
PDSA: Plan, do, study, act
WHO: World Health Organization
SOAP: Subjective, objective, assessment, and plan
NICE: National Institute for Health and Care Excellence
PRISMA: Preferred reporting items for systematic reviews and meta-analyses
