# Supplementary material for: Can Checklists Solve Our Ward Round Woes? A Systematic Review
Source: World J Surg. 2022 Jul 3;46(10):2355–64. doi: 10.1007/s00268-022-06635-5 (PMC9436887; doi:10.1007/s00268-022-06635-5)
Supplement: Supplementary file 1 — Supplementary file1 (DOCX 14 kb) [file 268_2022_6635_MOESM1_ESM.docx]

**Supplementary Figure 1. Full search strategy Embase, Medline and PyschInfo**

Database: Embase <1974 to 2021 August 17>

Search Strategy:

--------------------------------------------------------------------------------

1. (ward round* or clinical round* or teaching round* or patient round*).mp. (3298)

2. Teaching Round/ (810)

3. 1 or 2 (3298)

4. exp Documentation/ (341932)

5. (document* or template* or checklist* or proforma* or check-list* or ticklist* or tick-list*).mp. [mp=title, abstract, heading word, drug trade name, original title, device manufacturer, drug manufacturer, device trade name, keyword, floating subheading word, candidate term word] (814749)

6. 4 or 5 (1054004)

7. exp surgery/ (5127685)

8. (surgery or post-operative or postoperative).mp. (4019512)

9. 7 or 8 (5891923)

10. 3 and 6 and 9 (158)

11. limit 10 to yr="1980 -Current" (158)

***************************

Database: Ovid MEDLINE(R) and Epub Ahead of Print, In-Process, In-Data-Review & Other Non-Indexed Citations and Daily <1946 to August 17, 2021>

Search Strategy:

--------------------------------------------------------------------------------

1. (ward round* or clinical round* or teaching round* or patient round*).mp. (2514)

2. Teaching Rounds/ (1245)

3. 1 or 2 (2514)

4. Documentation/ (18283)

5. (document* or template* or checklist* or proforma* or check-list* or ticklist* or tick-list*).mp. [mp=title, abstract, original title, name of substance word, subject heading word, floating sub-heading word, keyword heading word, organism supplementary concept word, protocol supplementary concept word, rare disease supplementary concept word, unique identifier, synonyms] (588575)

6. 4 or 5 (588575)

7. exp Specialties, Surgical/ (207066)

8. (surgery or post-operative or postoperative).mp. (3001008)

9. 7 or 8 (3094065)

10. 3 and 6 and 9 (48)

11. limit 10 to yr="1980 -Current" (48)

***************************

Database: APA PsychInfo <1987 to August 17, 2021>

Search Strategy:

--------------------------------------------------------------------------------

1. (ward round* or clinical round* or teaching round* or patient round*).mp (258)

2. Teaching Rounds/ (0)

3. 1 or 2 258

4. Documentation/ (0)

5. (document* or template* or checklist* or proforma* or check-list* or ticklist* or tick-list*).mp. (179941)

6. 4 or 5 (179941)

7. (surgery or post-operative or postoperative).mp. [mp=title, abstract, heading word, table of contents, key concepts, original title, tests & measures, mesh] (34674(

8. exp Surgery/ (62401)

9. 7 or 8 (76827)

10. 3 and 6 and 9 (0)

11. limit 10 to yr=1980 -Current (0)
