# Supplementary material for: Can Checklists Solve Our Ward Round Woes? A Systematic Review
Source: World J Surg. 2022 Jul 3;46(10):2355–64. doi: 10.1007/s00268-022-06635-5 (PMC9436887; doi:10.1007/s00268-022-06635-5)
Supplement: Supplementary file 2 — Supplementary file2 (DOCX 17 kb) [file 268_2022_6635_MOESM2_ESM.docx]

**Supplementary Table 1. Excluded studies from full-text review**

| **Author** | **Journal** | **Title** | **Reason** |
| --- | --- | --- | --- |
| Alamri et al., 2016  {Alamri, 2016, Surgical ward round checklist: does it improve medical documentation? A clinical review of Christchurch general surgical notes} | ANZ Journal of Surgery | Surgical ward round checklist: does it improve medical documentation? A clinical review of Christchurch general surgical notes | Follow up study: no pre and post intervention |
| Blackburn et al., 2020 | European Geriatric Medicine | Assessment and prevention of venous thromboembolism in orthogeriatrics inpatients | Abstract only |
| Blucher et al., 2014 | ANZ Journal of Surgery | Ward safety checklist in the acute surgical unit | Wrong outcomes; did not observe documentation |
| Boland 2015 | BMJ Quality Improvement Reports | Implementation of a ward round pro-forma to improve adherence to best practice guidelines | Wrong outcomes: adherence to best practice guidelines |
| Dewson et al., 2020 | Postgraduate Medical Journal | Surgical ward round proforma can improve documentation and efficiency of ward rounds | Wrong outcomes: values not described |
| Ehsanullah et al., 2015 | Annals of Medicine and Surgery | The surgical admissions proforma: Does it make a difference? | Wrong study design: looking at admission documentation rather than ward rounds |
| Grey 2015 | BJU International | 'Registrar of the Week' and a bespoke ward round checklist optimise the quality of urology in-patient care | Abstract only |
| Hale et al., 2015 | BMJ Quality Improvement Reports | Developing a ward round checklist to improve patient safety | Wrong patient population: gastroenterology team |
| Kajouj et al., 2020 | British Journal of Surgery | Implementation of structured ward round documentation to enhance patients' care | Abstract only |
| Pucher et al., 2014 | British Journal of Surgery | Randomized clinical trial of the impact of surgical ward-care checklists on postoperative care in a simulated environment | Wrong setting: simulated environment |
| Pucher et al., 2014 | Journal of the American College of Surgeons | Surgical ward-care checklists improve postoperative care in a simulated environment: A randomized controlled trial | Abstract only |
| Read et al., 2021 | ANZ Journal of surgery | Ward round checklist improves patient perception of care | Wrong outcomes; did not measure documentation |
| Roberts et al., 2020 | British Journal of Surgery | A Multidisciplinary Patient Safety Checklist improves Medicines Reconciliation in a Tertiary Plastic Surgery Unit | Poster only |
| Shaikh et al., 2017 | International Journal of Surgery | Weekend and out of hours surgical handover (WOOSH): A checklist format improves efficiency and patient safety | Abstract only |
| Sproson et al., 2017 | International Journal of Surgery | Implementing a urological ward round patient safety checklist: A complete audit cycle | Abstract only |
| Thiede et al., 2010 | Zentralblatt fur Chirurgie - Zeitschrift fur Allgemeine, Viszeral- und Gefasschirurgie | Documentation and management of complications - Also document normal findings? | Not published in English |
| Thompson et al., 2004 | Postgraduate Medical Journal | Do post-take ward round proformas improve communication and influence quality of patient care? | Wrong Patient population: did not state if patients were surgical |
| Tranter-Entwistle et al., 2020 | ANZ Journal of surgery | Introduction and validation of a surgical ward round checklist to improve surgical ward round performance in a tertiary vascular service | Wrong outcomes: documentation not assessed |
| VanMil et al., 2016 | Obesity Facts | The postoperative checklist for bariatric surgery; which parameters should be used? | Abstract only |
| VanMil et al., 2015 | Surgery for Obesity and Related Diseases | The postoperative checklist for bariatric surgery | Abstract only |
| Wright et al., 2009 | Health Informatics Journal | Does a post-take ward round proforma have a positive effect on completeness of documentation and efficiency of information management? | Wrong patient population: mixture of medical and surgical population |
