# Supplementary material for: Can Checklists Solve Our Ward Round Woes? A Systematic Review
Source: World J Surg. 2022 Jul 3;46(10):2355–64. doi: 10.1007/s00268-022-06635-5 (PMC9436887; doi:10.1007/s00268-022-06635-5)
Supplement: Supplementary file 3 — Supplementary file3 (DOCX 34 kb) [file 268_2022_6635_MOESM3_ESM.docx]

| **Supplementary Table 2. Data Extraction Table** | | | | | | | | | |
| --- | --- | --- | --- | --- | --- | --- | --- | --- | --- |
| **Author**  **Year**  **Country**  **Journal**  **Specialty** | **Study Period** | **Type of Study** | **Aim** | **Number of participants** | **No. of Patients** | **Number of participants** | **Interventions** | **Checklist Education** | **Outcomes** |
| Al-Mahrouqi H et al.  2013  Christchurch, New Zealand  BMJ Quality Improvement Reports  General Surgery | 6 months - May 2012 to November 2012  •Baseline measurement: (1st - 8th May 2012) 1-week retrospective review of clinical notes •*Proforma introduced for 6 months to ensure teams were accustomed to its use* •Post-intervention measurement: 1 week in November 2012 | Single Centre Observational Pre- and Post-study | To improve documentation | Both study weeks included 5-6 different consultants | 108 Pre-intervention  103 Post-intervention | Both study weeks included 5-6 different consultants | Proforma introduced as stickers attached onto paper-based notes | Team given 6 months to practice proforma | A) Uptake of proforma: 78/103 (75.7%)  B) 1) Date and Time: 37% 🡪 72%* (p<0.01) 2) Signature: 87% 🡪 89% 3) Impression: 40% 🡪 61%* (p<0.01) 4) Dietary plan: 50% 🡪 60% 5) All of above: 13% 🡪 31%*  C) 1) Nurses unsure of dietary plan: 20% 🡪 12% 2) Nurse contacted the team for clarification: 12% 🡪 8% 3) Response rate for nurses using proforma: 89% |
| Banfield D et al.  2017  Bath, UK  BMJ Quality Improvement Reports  Acute Surgery | 3 years - April 2014 to February 2017  Cycles:  • Baseline April 2014 - 3 weeks  • 2nd Post intervention: June 2014 (Weekday and Weekend separated) AND October 2014  •Checklist amended, 3rd Post intervention: April 2015, February 2017 | Single Centre Observational Pre- and Post-study | To improve both communication and documentation on the surgical Post-take ward round, influencing patient safety | 7 Doctors Pre-intervention | Baseline: n=31 •Post 1st Intervention (PDSA 2): n=24 weekday, n=18 weekend in June 2014, 27 delayed in October 2014  •Post 2nd intervention (PDSA 3): n=26 April 2015 and n= 20 Feb 2017 | 7 Doctors Pre-intervention | Checklist printed and laminated, and kept in office | PDSA 1: Promotion of checklist via presentation given to all surgical consultants and registrars at monthly audit meeting  PDSA 2: Data presented to surgical consultants to encourage further use of checklists  PDSA 3: Checklist amended from feedback from users | A) Uptake not stated, but states not all consultants are using it  B) Safety parameters:  1) Examination  2) Observations  3) Impression: 60% 🡪 87.5% (weekends only)  4) Investigations: 60% 🡪 87.5% (weekends only)  5) Antibiotics  6) IV Fluids  7) VTE assessment  8) Nutritional status  9) Length of stay 🡪 drug chart review  10) Ceiling of treatment: 10% 🡪 50%  11) Mean compliance: ~40% 🡪 ~55% 🡪 ~55% 🡪 65% 🡪 78%  C) Initial survey (7 FY1 Drs) shows hesitancy to initiate checklist (60% unhappy), 80% had better understanding, 100% agree that it could help facilitate discussion regarding resuscitation status.  Follow up survey 3 years Post inception: 100% of FY1 (10), Consultants (3), Regs (4), Senior Nurses (4) agreed that it contributed positively to patient safety, some FY1 still reserved to initiate checklist |
| Dhillon P et al.  2011  Dublin, Ireland  Irish Medical Journal  General Surgery, Vascular Surgery, Plastic Surgery, and Neurosurgery | 8-day period; not stated when | Single Centre Cohort Study | To improve patient safety through better communication, improved handovers, and improved record keeping | 2x 5 randomly selected consultant led teams (control and study group) | •53 Control  •34 Intervention | 2x 5 randomly selected consultant led teams (control and study group) | Proforma as stickers vs No proforma/sticker | Members of both the study and control group educated on the importance of ward round handovers and the necessary components required to be documented- Educated on use of proforma | A) Safety Parameters:  1) Senior clinician: 25% 🡪 77%  2) Team Name: 43% 🡪 100%  3) Management Plan: 23% 🡪 77%  4) Abnormal Bloods: 15% 🡪 85%  5) Vitals: 27% 🡪 73%  B) Adherence to Good Surgical Practice Guidelines: 55% 🡪 91%  C) Pre-intervention 38 doctors interviewed:  1) 31 believed documentation could be improved  2) 76% thought their documentation of clinical findings and plan was complete  3) 18 thought handovers could be improved with checklist, 12 were undecided  D) Team details documented - Senior Clinician: 25% 🡪 77%, Team name: 43% 🡪 100% |
| Dolan R and Broadbent P  2016  Glasgow, UK  Annals of Medicine and Surgery  Acute Surgery | Initial audit, then reaudit after 7-day period of proforma introduction | Single Centre Observational Pre- and Post-study | To assess if use of a standardised proforma can be effective in improving documentation | Presented to everyone; however not stated who participated | •50 Pre-intervention  • 47 Post-intervention | Presented to everyone; not stated who participated | Ward round proforma placed in admission document and used for Post-take ward rounds. | Disseminated information about introduction of ward round proforma- Oral presentation about the study and foundation of doctor teaching | A) Safety Parameters:  1) Patient name/Chi Sticker: 96%🡪 100%  2) Subjective: 84% 🡪 100%*  3) Objective/Examination: 48% 🡪 100%*  4) Impression/Diagnosis: 30% 🡪 98%*  5) Plan: 98% 🡪 100%  6) Diet: 16% 🡪 83%*  7) Discharge: 14% 🡪 16%*  8) Discharge planning: 20% 🡪 40%*  9) Signature: 96% 🡪 100%  10) Grade: 62% 🡪 100%*  B) Staff satisfaction with proforma: 94% junior staff, 85% middle grade, 86% consultants |
| Gilliland N et al.  2018  Bristol, UK  BMJ Quality Improvement Reports  Urology | PDSA 1: template used for 1 month 🡪 1 random day selected to measure compliance  PDSA 2: 1 random day chosen 1 month after introduction of template to review  PDSA 3: 1 random day one month after introduction of updated template | Single Centre Observational Pre- and Post-study | To improve patient safety, compliance to CQUIN targets, and ward round documentations by using a developed standardised paper-based ward round template | Given to different members of multi-disciplinary team but no numbers or indication where from | •14 Cycle 1 (n=14)  •17 Cycle 2 (n=17)  •14 Cycle 3 (n=14) | Given to different members of multi-disciplinary team but no numbers or indication where from | Proforma printed onto paper and used on ward rounds | Not stated if any education on template, but feedback after each cycle to amend it | A) Uptake: Cycle 2 60%, Cycle 3 65%  B) Safety parameters:  1) Observations: 64% 🡪 90% 🡪 100%  2) NEWS 7: 60% 🡪 100%  3) VTE prophylaxis: 29% 🡪 67% 🡪 92%  4) Antibiotics: 0% 🡪 100% 🡪 100%  5) Fluid balance: 21% 🡪 60% 🡪 100%  6) DNAR/TEP: 29% 🡪 50% 🡪 78% |
| Krishnamohan N et al.  2019  UK  Journal of Multidisciplinary Healthcare  Surgical (including urology and vascular surgery) | •8 Months (April 2015 to August 2016) 16 months  •December 2016 if counting note review | Single Centre Observational Pre- and Post-study + Extension | Implementation of surgical WR checklist for daily surgical WR to improve patient safety and documentation | Stated that it was provided to all nursing staff and doctors, but exact numbers not stated | •Baseline 72  •Post implementation: 61 | Stated that it was provided to all nursing staff and doctors; exact numbers not stated | Stickers placed within clinical entry and completed following assessment, review, and documentation of each of the parameters. | Information regarding the WR checklist provided to nursing staff and Junior doctors via clinical governance meetings and teaching sessions | A) Uptake: Not stated  B) Safety parameters:  1) VTE Prescription: 28% vs 74%  2) Antibiotics: 35% vs 74%  3) Fluid balance: 8% vs 76%  4) Blood tests: 23% vs 80%  5) Patient observations: 34% vs 87%  6) Drug cardex: 25% vs 85%  7) Estimated Date of Discharge  8) Overall: 26% vs 79%, Re audit at 3 months: 72%  C) Reported adverse effects Pre- vs Post-checklist:  1) Prescription errors: 13 vs 2  2) Antibiotic errors: 4 vs 0, Fluid balance errors: 2 vs 0  3) Patient observation chart errors: 2 vs 0  4) VTE cases: 11 vs 10 |
| Ng J et al.  2018  UK  BMJ Quality Improvement Reports  General Surgery | 6 months (September 2016 to March 2017)  Baseline - September 2016 PDSA 1 - January 2017 (3 days)  PDSA 2 - March 2017 (3 days) | Single Centre Observational Pre- and Post-study/ Cross-sectional study | To design a user-friendly, clear, unambiguous ward round stick, to gather feedback on the usefulness of the sticker, to measure and compare quality of surgical ward round against recommended standards with or without sticker, to implement and educate surgical teams on the use of ward round stick as an adjunct to good patient care | No description of number of doctors | •109 baseline  •71 First cycle  •76 Second cycle | No description of number of doctors | Introduction of a ward round sticker to be used in ward rounds | Clinical members of general surgery. Made aware of the stickers via email, no education of its use | A) Uptake: Cycle 1: 51 with sticker vs 20, Cycle 2: 40 with sticker vs 36  B) Safety Parameters (BL vs No sticker vs With Sticker):  1) Date: 98% vs 100% vs 100%  2) Time: 85% vs 100% vs 96%  3) Led by: 95% vs 100% vs 100%  4) Remarks: 91% vs 100% vs 100%  5) Observations: 64% vs 60% vs 100%*  6) On Examination: 41% vs 70% vs 80%  7) Drugs: 6% vs 45% vs 92%*  8) IV: 4% vs 25% vs 92%*  9) Analgesia: 2% vs 15% vs 90%*  10) Anti-emetics: 0% vs 0% vs 90%*  11) Enoxaparin: 0% vs 5% vs 96%*  12) TEDs: 0% vs 0% vs 94%*  13) Impression/Diagnosis: 32% vs 40% vs 69%*  14) Plan: 96% vs 95% vs 100%  15) Signature: 95% vs 95% vs 98%  16) Bleep: 68% vs 90% vs 96%  17) GMC number: 10% vs 57%  18) Grade: 10% vs 94%  C) Safety Parameters (Cycle 2 Sticker (40) vs No sticker (36)):  1) Impression/Diagnosis: 39% no sticker, 80% with sticker*  2) NEWS: 50% vs 92.5%*  3) Drug Chart: 0.05% vs 80%*  4) Fluid therapy: 0.05% vs 85%*  5) Analgesia: 0.05% vs 75%*  6) Anti-emetic: 0% vs 67%*  7) Antibiotic: 0.08% vs 77.5%*  8) VTE Form: 87.5% vs 0% vs 87.5%*  9) Enoxaparin: 0.03% vs 75%*  10) TEDs: 0.03% vs 77.5%*  11) Dietary status: 17% vs 67.5%  12) Bloods: 11% vs 62.5%  13) Nurse: 0% vs 65%  14) Outstanding bloods, CT MRI, USS, Scope, Referral, Other *****  15) Stamp: 0% vs 12.5%  16) GMC Number: 11% vs 35%  17) Bleep: 72% vs 87.5%  18) Grade: 14% vs 67.5%  19) Signature: 75% vs 90%  D) Feedback - Cycle 1: 68% felt sticker made WR better, 64% felt efficiency of patient care was better, 86% felt identifying patients issues better, 59% felt patient care and management was better |
| Pitcher M et al.  2015  Australia  ANZ Journal of Surgery  General Surgery | Initial Phase & follow up phase: 2-week period each.  However, no indication of time between control and study group | Single Centre Observation Pre- and Post-study | Not stated | 3-day registrars, 1 night registrar and 3 interns | •50 baseline  •50 Post-intervention | 3-day registrars, 1 night registrar and 3 interns | Introduction of a structured surgical ward round progress sheet | Not Stated | A) Uptake: Not stated  B) Safety parameters:  1) Date/Time: 100% vs 100 / 74% vs 88%  2) Presence of Nurse: 0% vs 78%*  3) History: 98% vs 98%  4) Examination: 62% vs 100%*  5) Hand hygiene: 0% vs 86%*  6) Observations: 88% vs 100%*  7) Pain: 8% vs 86%*  8) Cannulas: 4% vs 78%*  9) Nutrition: 46% vs 82%*  10) DV prophylaxis: 12% vs 76%*  11) Urine/IDC: 6% vs 76%*  12) Wounds/Drains: 14% vs 58%*  13) Drug Chart: 2% vs 64%*  14) Bowel Chart: 0% vs 24%*  15) Fluid balance Chart: 6% vs 48%*  16) Plan: 90% vs 100%*  17) Bloods: 18% vs 18%  18) Radiology: 6% vs 34%*  19) Care limits: 0% vs 0%  20) Referrals: 10% vs 56%*  21) Discharge: 34% vs 82%*  22) Signature/Name/Contact: 100% vs 100%  23) Designation: 8% vs 98%* |
| Talia A et al.  2017  Australia  Orthopedics  Orthopaedics | 4 weeks | Single centre cohort study | To build on idea of using a structured checklist and apply it to aspects of day-to-day surgical routine to minimise human error and missed aspects of care | Same observers (2) recorded all 4 weeks of observation- Same junior medical staff involved in both cohorts - 4 junior medical staff members observed in cohort a and cohort b | 132 Patients for Standard care, 68 Patients for structured WR checklist | Same observers (2) recorded all 4 weeks of observation- Same junior medical staff involved in both cohorts - 4 junior medical staff members observed in cohort a and cohort b | Structured checklist printed and educated | Junior medical staff educated about checklist and given 1 week to practice using it | 1) Time Spent Per Patient: 93.2s vs 77.8s  2) Pre- and Post-operative status: 42.4% vs 76.5%*  3) Fasting Status: 9.1% vs 70.6%*  4) Surgical details: 38.6% vs 85.3%*  5) Vital signs: 83.3% vs 89.7%  6) Physical examination: 31.1% vs 50%*  7) Wound observations: 28% vs 60.3%*  8) Wound or dressing plan: 6.8% vs 92.6%*  9) VTE prophylaxis plan: 6.8% vs 92.6%  10) Bowels and aperients: 40.9% vs 91.2%*  11) Weight bearing status: 11.4% vs 83.8%*  12) Ongoing management plan: 93.9% vs 98.5% |
