# Supplementary material for: Can Checklists Solve Our Ward Round Woes? A Systematic Review
Source: World J Surg. 2022 Jul 3;46(10):2355–64. doi: 10.1007/s00268-022-06635-5 (PMC9436887; doi:10.1007/s00268-022-06635-5)
Supplement: Supplementary file 4 — Supplementary file4 (DOCX 23 kb) [file 268_2022_6635_MOESM4_ESM.docx]

**Supplementary Table 3. Average change in documentation pre-intervention to final intervention (all outcomes)**

| **Outcomes** | **Al-Mahrouqi**  **2013** | **Banfield**  **2017** | **Dhillon**  **2011** | **Dolan**  **2016** | **Gilliland**  **2018** | **Krishnamohan**  **2019** | **Ng**  **2018** | **Pitcher**  **2015** | **Talia**  **2017** |
| --- | --- | --- | --- | --- | --- | --- | --- | --- | --- |
| **Patient Name** |  |  |  | 4% |  |  |  |  |  |
| **Date & Time** | 35% |  |  |  |  |  | 2% |  |  |
| **Team Name** |  |  | 57% |  |  |  | 5% |  |  |
| **Senior Clinician** |  |  | 52% |  |  |  |  |  |  |
| **Signature** | 2% |  |  | 4% |  |  | -5% |  |  |
| **Grade** |  |  |  | 38% |  |  | 58% |  |  |
| **GMC NUMBER** |  |  |  |  |  |  | 25% |  |  |
| **Presence of nurse** |  |  |  |  |  |  | 65% |  |  |
| **Surgical Details** |  |  |  |  |  |  |  |  | 47% |
| **Hand Hygiene** |  |  |  |  |  |  |  |  |  |
| **History** |  |  |  | 67% |  |  |  |  | 34% |
| **Subjective** |  |  |  | 16% |  |  |  |  | 34% |
| **Examination** |  |  |  | 52% |  |  | 39% |  | 19% |
| **Pain** |  |  |  |  |  |  |  |  |  |
| **NEWS** |  |  |  |  | 93% |  | 43% |  |  |
| **Observations/Vitals** |  |  | 46% |  | 36% | 53% | 36% | 53% | 6% |
| **Cannulas** |  |  |  |  |  |  | 88% |  |  |
| **Wounds/drains** |  |  |  |  |  |  |  |  | 32% |
| **Antibiotics** |  |  |  |  | 100% | 39% | 77% | 39% |  |
| **Bloods** |  |  | 70% |  |  | 57% | 52% | 57% |  |
| **Analgesia** |  |  |  |  |  |  | 73% |  |  |
| **Antiemetics** |  |  |  |  |  |  | 67% |  |  |
| **Drug Chart Review** |  |  |  |  |  | 60% | 74% | 60% |  |
| **Fluid** |  |  |  |  | 79% | 68% | 85% | 68% |  |
| **Urine/IDC** |  |  |  |  |  |  |  |  |  |
| **Bowel Chart** |  |  |  |  |  |  |  |  | 50% |
| **Radiology** |  |  |  |  |  |  |  |  |  |
| **Investigations** |  |  |  |  |  |  |  |  |  |
| **VTE assessment** |  |  |  |  | 63% | 46% |  | 46% | 86% |
| **TEDS** |  |  |  |  |  |  | 78% |  |  |
| **Enoxaparin** |  |  |  |  |  |  | 75% |  |  |
| **Impression** | 21% |  |  | 52% |  |  | 48% |  |  |
| **Management Plan** |  |  | 54% | 2% |  |  | 2% |  | 6% |
| **Dietary Plan** | 10% |  |  | 67% |  |  | 51% |  | 62% |
| **Wound or dressing plan** |  |  |  |  |  |  |  |  | 86% |
| **Weight bearing Plan** |  |  |  |  |  |  |  |  | 72% |
| **Referrals to be made** |  |  |  |  |  |  |  |  |  |
| **Ceiling of care** |  |  |  |  | 49% |  |  |  |  |
| **EDD** |  |  |  | 20% |  |  |  |  |  |
| **Discharge** |  |  |  | 2% |  |  |  |  |  |
| **Overall** | 18% |  |  |  |  | 53% |  | 53% |  |
