# Supplementary material for: Can Checklists Solve Our Ward Round Woes? A Systematic Review
Source: World J Surg. 2022 Jul 3;46(10):2355–64. doi: 10.1007/s00268-022-06635-5 (PMC9436887; doi:10.1007/s00268-022-06635-5)
Supplement: Supplementary file 5 — Supplementary file5 (DOCX 17 kb) [file 268_2022_6635_MOESM5_ESM.docx]

| **Supplementary Table 4. Risk of Bias Outcomes using Newcastle Ottawa Scale** | | | | | | | | | | |
| --- | --- | --- | --- | --- | --- | --- | --- | --- | --- | --- |
| **Study** | **Selection** | | | | **Comparability** | **Outcome** | | | **Total points** | |
|  | **Representativeness of the exposed cohort** | **Selection of the non-exposed cohort** | **Ascertainment of exposure** | **Demonstration that outcome of interest was not present at the start of study** | **Comparability of cohorts on the basis of the design or analysis** | **Assessment of outcomes** | **Was follow-up long enough for outcomes to occur** | **Adequacy of follow up cohorts** |  |  |
| Al-Mahrouqi H et al., 2013 | 1 | 0 | 1 | 0 | 1 | 1 | 1 | 1 | 6 | ****** |
| Banfield D et al., 2017 | 0 | 0 | 0 | 0 | 0 | 1 | 1 | 1 | 3 | *** |
| Dhillon P et al., 2011 | 1 | 1 | 1 | 0 | 0 | 1 | 1 | 1 | 6 | ****** |
| Dolan R and Broadbent P, 2016 | 0 | 0 | 1 | 0 | 0 | 1 | 1 | 1 | 4 | **** |
| Gilliland N et al., 2018 | 0 | 0 | 1 | 0 | 0 | 1 | 1 | 1 | 4 | **** |
| Krishnamohan N et al., 2019 | 0 | 0 | 1 | 0 | 0 | 1 | 1 | 1 | 4 | **** |
| Ng J et al., 2018 | 0 | 0 | 1 | 0 | 0 | 1 | 1 | 1 | 4 | **** |
| Pitcher M et al., 2015 | 0 | 0 | 1 | 0 | 1 | 1 | 1 | 1 | 5 | ***** |
| Talia A et al., 2017 | 1 | 1 | 0 | 0 | 1 | 1 | 1 | 1 | 6 | ****** |
